# Prognosis of activities of daily living function in hospitalized patients with nursing and healthcare‐associated pneumonia due to COVID‐19

**DOI:** 10.1111/irv.13045

**Published:** 2022-09-17

**Authors:** Naoyuki Miyashita, Yasushi Nakamori, Makoto Ogata, Naoki Fukuda, Akihisa Yamura

**Affiliations:** ^1^ First Department of Internal Medicine, Division of Respiratory Medicine, Infectious Disease and Allergology Kansai Medical University Hirakata Japan; ^2^ Department of Emergency Medicine Kansai Medical University Medical Center Moriguchi Japan

**Keywords:** COVID‐19, nursing and healthcare‐associated pneumonia, physical function, prognosis, SARS‐CoV‐2

## Abstract

Nursing and healthcare‐associated pneumonia (NHCAP) is associated with decreased physical function. We investigated the functional outcomes at 1 year after hospital discharge in patients with COVID‐19 pneumonia. Functional decline rates for calculating the Barthel Index at the time of hospital discharge and at 1 year after hospital discharge were significantly higher in the NHCAP group than the community‐acquired pneumonia group (at hospital discharge, 54.0% vs. 31.2%, respectively, *p* < 0.0001; 1 year follow‐up, 37.9% vs. 8.6%, respectively, *p* < 0.0001). It is necessary to consider early rehabilitation, and treatment depending on the presence or absence of applicable criteria for NHCAP.

## 1 INTRODUCTION

Pneumonia remains a significant cause of morbidity and death worldwide despite the availability of potent antibiotic therapies. For the management of pneumonia in elderly patients, nursing home residents, and disabled persons, the Japan Respiratory Society (JRS) guidelines defined a new pneumonia category as nursing and healthcare‐associated pneumonia (NHCAP) separate from community‐acquired pneumonia (CAP).^1^ Rates of intensive care unit (ICU) stay or in‐hospital mortality were significantly higher in patients with NHCAP than those with CAP.^2^, ^3^, ^4^

In countries with aging populations or with a large number of elderly persons, physicians should emphasize activities of daily living (ADL) function rather than mortality as a prognostic assessment. Functional decline during or after hospitalization is associated with adverse health outcomes, prolonged hospital stays due to more frequent occurrences of hospital complications, and more frequent episodes of early hospital admission.^5^, ^6^, ^7^, ^8^, ^9^ Our previous study that focused on physical outcomes after hospital discharge in patients with pneumonia demonstrated that rates of decline in ADL function were significantly higher in NHCAP patients than CAP patients.^10^

The novel severe acute respiratory syndrome coronavirus 2 (SARS‐CoV‐2) causes frequent outbreaks in facilities, such as welfare facilities for persons with disabilities and long‐term care health facilities, that meet the criteria for NHCAP. In this study, we investigated the deterioration of physical function in NHCAP patients and compared it with CAP patients.

## 2 PATIENTS AND METHODS

### 2.1 Study populations

The present study was conducted at five institutions (Kansai Medical University Hospital, Kansai Medical University Medical Center, Kansai Medical University Kori Hospital, Kansai Medical University Kuzuha Hospital, and Kansai Medical University Temmabashi General Clinic) between February 2020 and June 2021. COVID‐19 was diagnosed with positive reverse transcription polymerase chain reaction results from sputum or nasopharyngeal swab specimens in accordance with the protocol recommended by the National Institute of Infectious Diseases, Japan.

NHCAP was defined as pneumonia acquired in the community with one or more of the following risk factors^1^: Group (A) pneumonia diagnosed in a resident of an extended care facility, long‐term care health facility, or psychiatric hospital; Group (B) pneumonia diagnosed in a person who had been discharged from a hospital within the preceding 90 days; Group (C) pneumonia diagnosed in an elderly or disabled person who is receiving nursing care with an Eastern Cooperative Oncology Group performance status (PS) of 3 or 4; and Group (D) pneumonia diagnosed in a person who is receiving regular endovascular treatment as an outpatient (dialysis, antibiotic therapy, chemotherapy, and immunosuppressant therapy). During the study period, 840 patients with COVID‐19 pneumonia were recognized. Of these, we finally enrolled 124 hospitalized patients with NHCAP and 314 hospitalized patients with CAP that we could follow‐up for 1 year. Informed consent was obtained from all patients, and the study protocol was approved by the Ethics Committee of Kansai Medical University (approval number 2020319).

### 2.2 Evaluation of functional outcomes

The ADL assessment for calculating the Barthel Index consisted of the following 10 indices: feeding; bathing; grooming; dressing; bowels; bladder; toilet use; transfers; morbidity; and stairs. In the present study, we calculated the difference in ADL scores between baseline (1 week before admission), at hospital discharge, and 1 year after discharge from our hospitals. The difference was categorized into two groups: declined (≥1) and not declined (0). Of the pneumonia cases, we excluded bedridden cases because these patients were not able to change their ADL score between before and after admission to hospital.

### 2.3 Statistical analysis

Statistical analysis was performed using Stat View version 5.0. (SAS Institute Inc, Cary, NC, USA). The incidence of clinical findings was analyzed using Fisher's Exact test. Continuous variables were compared using the Student's *t* test when variables were normally distributed, and the Mann–Whitney U test was used when variables were non‐normally distributed.

## 3 RESULTS

### 3.1 Functional decline in the CAP group and NHCAP group

Patients with NHCAP were significantly older than those with CAP (73 vs. 80 years old, *p* < 0.0001), but the male/female ratio did not differ between the two groups (Table 1). The incidences of some co‐morbid illnesses were significantly higher in patients with NHCAP than those with CAP. Functional decline rates for calculating the Barthel Index at the time of hospital discharge and at 1 year after hospital discharge were significantly higher in the NHCAP group than the CAP group (at hospital discharge, 31.2% vs. 54.0%, respectively, *p* < 0.0001; 1 year follow‐up, 8.6% vs. 37.9%, respectively, *p* < 0.0001). Of 67 NHCAP patients who declined in physical function at the time of hospital discharge, 47 patients (70.1%) still showed functional decline at 1 year later.

**TABLE 1:** Clinical characteristics and outcomes of patients with COVID‐19 pneumonia between the community‐acquired pneumonia group and the nursing and healthcare‐associated pneumonia group

Variables	Community‐acquired pneumonia	Nursing and healthcare‐associated pneumonia	*p* value
No. of patients	314	124	
Median age (IQR), years	68 (73–78)	80 (72–84)	<0.0001
No. of males/females	208/106	73/51	0.1522
No. (%) of patients with comorbid illnesses			
Diabetes mellitus	88 (28.0)	31 (25.0)	0.5530
Chronic lung disease	51 (16.2)	19 (15.3)	0.8855
Chronic heart disease	27 (8.6)	26 (21.0)	0.0009
Neoplastic disease	21 (6.7)	19 (15.3)	0.0089
Cerebrovascular disease	21 (6.7)	24 (19.4)	0.0002
Chronic renal disease	14 (4.59)	23 (18.5)	<0.0001
Chronic liver disease	10 (3.2)	7 (5.6)	0.2719
Autoimmune disease	11 (3.5)	4 (3.2)	>0.9999
No. (%) of patients with deterioration of physical activity			
At hospital discharge	98 (31.2)	67 (54.0)	<0.0001
At 1 year after hospital discharge	27 (8.6)	47 (37.9)	<0.0001
Barthel index (average)			
Before admission to hospitals	94	58	<0.0001
At hospital discharge	38	24	<0.0001
At 1 year after hospital discharge	77	36	<0.0001
No. (%) of patients with in‐hospital mortality	14 (4.5)	31 (25.0)	<0.0001

*Note*: Continuous values are presented as medians and interquartile ranges (IQRs) and categorical/binary values as counts and percentages.

### 3.2 Functional decline in different NHCAP groups

Functional decline rates at 1 year after hospital discharge were higher in Group A (44.1%) and Group C (44.1%) than in Group D (33.3%), but these differences were not statistically significant (Table 2). Of 36 patients with poor PS in Group C who showed a decline in physical function at the time of hospital discharge, 30 patients (83.3%) still showed functional decline at 1 year later.

**TABLE 2:** Clinical characteristics and outcomes of patients with COVID‐19 pneumonia divided by different nursing and healthcare‐associated groups^a^

Variables	Group A	Group B	Group C	Group D
No. of patients	59	4	68	33
Median age (IQR), years	81 (73–85)	76 (71–80)	82 (76–86)	76 (70–82)
No. of males/females	33/26	4/0	33/35	21/12
No. (%) of patients with comorbid illnesses				
Diabetes mellitus	11 (18.6)	3	13 (19.1)	12 (36.4)
Chronic lung disease	9 (15.3)	1	12 (17.6)	4 (12.1)
Chronic heart disease	13 (22.0)	2	14 (20.6)	8 (24.2)
Neoplastic disease	1 (1.7)	1	4 (5.9)	14 (42.4)
Cerebrovascular disease	9 (15.3)	2	16 (23.5)	4 (12.1)
Chronic renal disease	3 (5.1)	0	3 (4.4)	19 (57.6)
Chronic liver disease	3 (5.1)	0	4 (5.9)	2 (6.1)
Autoimmune disease	2 (3.4)	0	4 (5.9)	0
No. (%) of patients with deterioration of physical activity				
At hospital discharge	38 (64.4)	1	36 (52.9)	18 (54.5)
At 1 year after hospital discharge	26 (44.1)	1	30 (44.1)	11 (33.3)
Barthel index (average)				
Before admission to hospitals	50	80	44	76
At hospital discharge	16	40	11	42
At 1 year after hospital discharge	28	40	18	61
No. (%) of patients with in‐hospital mortality	10 (16.9)	1	18 (26.5)	9 (27.3)

*Note*: Continuous values are presented as medians and interquartile ranges (IQRs) and categorical/binary values as counts and percentages.

^a^ Including overlapping cases.

## 4 DISCUSSION

Several studies demonstrated that early physiotherapy may prevent decline in ADL in elderly patients with pneumonia.^11^, ^12^, ^13^ Yagi et al assessed the effect of early rehabilitation on improving ADL in elderly patients with aspiration pneumonia.^11^ They showed that the early rehabilitation group exhibited significant improvement in ADL (OR 1.57, 95% CI 1.50–1.64, *p* < 0.001). Kim et al examined whether physical therapy for hospitalized older adults is associated with functional changes and early hospital readmission rate.^12^ Hospital‐based physical therapy had benefits in reducing the 30‐day hospital readmission rate of acutely ill older adults with CAP and declining physical function. Chigira et al also demonstrated the early physiotherapy may shorten the intensive care unit admission period and prevent decline in ADL in elderly patients.^13^ In our hospitals, patients with pneumonia usually took part in some form of physical rehabilitation program administered by physicians or physical therapists within 7 days after hospitalization. However, this program was not able to be applied for early rehabilitation in the COVID‐19 ward. Thus, an early physical rehabilitation program was developed in our hospitals from June 2021.

In April 2021, vaccination against SARS‐CoV‐2 was started in elderly people, and infection has subsequently shifted from elderly people to younger age groups in Japan. Because the number of severely ill patients and deaths due to COVID‐19 in elderly persons were markedly reduced, giving priority for SARS‐CoV‐2 vaccination to elderly people and people with comorbid illnesses was thought to be reasonable. However, it has not been evaluated whether SARS‐CoV‐2 vaccination improves functional decline or whether SARS‐CoV‐2 vaccination maintains physical activity after COVID‐19 pneumonia.

Our study had several limitations. Our study was performed before the SARS‐CoV‐2 vaccination period. From June 2021, the fifth wave of COVID‐19 began with a new lineage of SARS‐CoV‐2, the Delta variant, which spread rapidly throughout Japan. To clarify the effect of vaccination in relation to physical activity, a comparative study between vaccinated patients and non‐vaccinated patients with the Delta variant group is needed. Cognitive impairment is associated with adverse health outcomes after hospitalization. Several studies demonstrated older patients hospitalized for pneumonia are at risk of new‐onset cognitive impairment.^14^ Additionally, regarding COVID‐19, there is growing concern about possible cognitive consequences, with reports of “Long COVID” symptoms persisting into the chronic phase and case studies revealing neurological problems in severely affected patients.^15^ In this study, we were unable to evaluate cognitive function before and after COVID‐19 pneumonia. A further study focused on changes in cognitive function after COVID‐19 pneumonia is needed.

In conclusion, our results demonstrated that among patients with NHCAP, especially those with poor PS who showed a decline in function at hospital discharge, over 70% of these patients still showed functional decline at 1 year after hospital discharge. It is necessary to consider early rehabilitation, prevention, and treatment depending on the presence or absence of applicable criteria for NHCAP.

## CONFLICT OF INTEREST

The authors declare that they have no competing interests.

## ETHICS STATEMENT

The study protocol was approved by the Ethics Committee at Kansai Medical University and all participating facilities. Informed consent was obtained from all individual participants in the study.

## AUTHOR CONTRIBUTIONS

**Naoyuki Miyashita:** Conceptualization; data curation; investigation. **Yasushi Nakamori:** Data curation; investigation. **Makoto Ogata:** Formal analysis; investigation; methodology. **Naoki Fukuda:** Formal analysis; investigation; methodology. **Akihisa Yamura:** Data curation; formal analysis; methodology.

### PEER REVIEW

The peer review history for this article is available at https://publons.com/publon/10.1111/irv.13045.

## Data Availability

The data will not be shared because of participant confidentiality.

## References

[1] Kohno S , Imamura Y , Shindo Y , et al. Clinical practice guidelines for nursing‐ and healthcare‐associated pneumonia (NHCAP) [complete translation]. Respir Investig. 2014;51(2):103‐126. doi:10.1016/j.resinv.2012.11.001 23790739

[2] Ishida T , Tachibana H , Ito A , Yoshioka H , Arita M , Hashimoto T . Clinical characteristics of nursing and healthcare‐associated pneumonia: a Japanese variant of healthcare‐associated pneumonia. Intern Med. 2012;51(18):2537‐2544. doi:10.2169/internalmedicine.51.7987 22989823

[3] Fukuyama H , Yamashiro S , Tamaki H , Kishaba T . A prospective comparison of nursing‐ and healthcare‐associated pneumonia (NHCAP) with community‐acquired pneumonia (CAP). J Infect Chemother. 2013;19(4):719‐726. doi:10.1007/s10156-013-0557-1 23354936

[4] Kaku N , Nagashima S , Fukuda M , et al. The definition of healthcare‐associated pneumonia (HCAP) is insufficient for the medical environment in Japan: a comparison of HCAP and nursing and healthcare‐associated pneumonia (NHCAP). J Infect Chemother. 2013;19(1):70‐76. doi:10.1007/s10156-012-0454-z 22850821

[5] Sutton M , Grimmer‐Somers K , Jeffries I . Screening tools to identify hospitalized elders at risk of functional decline: a systematic review. Int J Clin Pract. 2008;62(12):1900‐1909. doi:10.1111/j.1742-1241.2008.01930.x 19166437

[6] Gill TM , Allore HG , Gahbauer EA , Murphy TE . Change in disability after hospitalization or restricted activity in older persons. Jama. 2010;304(17):1919‐1928. doi:10.1001/jama.2010.1568 21045098 PMC3124926

[7] Mudge AM , O'Rourke P , Denaro CP . Timing and risk factors for functional changes associated with medical hospitalization in older patients. J Gerontol a Biol Sci Med Sci. 2010;65(8):866‐872. doi:10.1093/gerona/glq069 20494952

[8] Buurman BM , Hoogerduijn JG , de Haan RJ , et al. Geriatric conditions in acutely hospitalized older patients: prevalence and one‐year survival and functional decline. PLos One. 2011;6(11):e26951. doi:10.1371/journal.pone.0026951 22110598 PMC3215703

[9] Buurman BM , Hoogerduijn JG , van Gemert EA , de Haan RJ , Schuurmans MJ , de Rooij SE . Clinical characteristics and outcomes of hospitalized older patients with distinct risk profiles for functional decline. PLos One. 2012;7(1):e29621. doi:10.1371/journal.pone.0029621 22238628 PMC3251572

[10] Kato T , Miyashita N , Kawai Y , et al. Changes in physical function after hospitalization in patients with nursing and healthcare‐associated pneumonia. J Infect Chemother. 2016;22(10):662‐666. doi:10.1016/j.jiac.2016.06.005 27493023

[11] Yagi M , Yasunaga H , Matsui H , et al. Effect of early rehabilitation on activities of daily living in patients with aspiration pneumonia. Geriatr Gerontol Int. 2016;16(11):1181‐1187. doi:10.1111/ggi.12610 26460175

[12] Kim SJ , Lee JH , Han B , et al. Effects of hospital‐based physical therapy on hospital discharge outcomes among hospitalized older adults with community‐acquired pneumonia and declining physical function. Aging Dis. 2015;6(3):174‐179. doi:10.14336/AD.2014.0801 26029475 PMC4441399

[13] Chigira Y , Takaki T , Igusa H , Dobashi K . Effects early physiotherapy with respect to severity of elderly patients admitted to an intensive care unit: a single center study in Japan. J Phys Ther Sci. 2015;27(7):2053‐2056. doi:10.1589/jpts.27.2053 26311924 PMC4540815

[14] Shah FA , Pike F , Alvarez K , et al. Bidirectional relationship between cognitive function and pneumonia. Am J Respir Crit Care Med. 2013;188(5):586‐592. doi:10.1164/rccm.201212-2154OC 23848267 PMC3827700

[15] Cohen K , Ren S , Heath K , et al. Risk of persistent and new clinical sequelae among adults aged 65 years and older during the post‐acute phase of SARS‐CoV‐2 infection: retrospective cohort study. BMJ. 2022;376:e068414. 35140117 10.1136/bmj-2021-068414 PMC8828141

